# A new association of Oculoauriculovertebral spectrum and persistent fifth aortic arch -double lumen aorta: a case report

**DOI:** 10.1186/s12887-022-03137-0

**Published:** 2022-02-21

**Authors:** İsmail Balaban, Meltem Ceyhan Bilgici, Kemal Baysal

**Affiliations:** 1 Department of Pediatric Cardiology, Yeni Yüzyıl University Faculty of Medicine, Merkez Mahallesi Çukurçeşme Caddesi No:51 Gaziosmanpaşa, İstanbul, Turkey; 2 Department of Pediatric Radiology, Ondokuz Mayıs University Faculty of Medicine, Samsun, Turkey; 3 Department of Pediatric Cardiology, Ondokuz Mayıs University Faculty of Medicine, Samsun, Turkey

**Keywords:** Oculoauriculovertebral spectrum, Persistent fifth aortic arch, Case report

## Abstract

**Background:**

Oculo-auriculo-vertebral spectrum is a heterogeneous group of genetic disorder, also known as Goldenhar Syndrome, which has several phenotypic features including craniofacial anomalies, cardiac, vertebral and central nervous system defects. Cardiovascular anomalies include ventricular septal defects, atrial septal defects, patent ductus arteriosus, Tetralogy of Fallot, double outlet right ventricle, aberrant right subclavian artery, coarctation of aorta, transposition of the great arteries, double inlet left ventricle, cor triatriatum, pulmonary artery stenosis, aortic stenosis, persistent left superior vena cava, partially or totally abnormal pulmonary venous return and bicuspid aortic valve. Persistent fifth aortic arch, also named as double lumen aortic arch, is a very rare cardivascular anomaly and usually associate other cardiac defects.

**Case presentation:**

We present a 7 month old patient with oculo-auriculo-vertebral spectrum signs as facial asymmetry, short neck, choanal atresia, cleft palate, bilateral preauricular skin tags, bilateral hypoplastic ear lobes, epibulbar dermoid cyst, rib, vertebrae and cardiovascular anomalies. Cardiovascular anomalies detected with echocardiography and computed tomography were malalignment ventricular septal defect and double lumen aorta, known as persistent fifth aortic arch.

**Conclusion:**

Various cardiovascular anomalies may accompany Goldenhar Syndrome. We present a case with persistent fifth aortic arch and Oculo-auriculo-vertebral spectrum and this is a new association that was not reported before in the literature.

## Background

Oculo-auriculo-vertebral spectrum (OAVS) (MIM 164210), also known as Goldenhar syndrome or hemifacial microsomia, is a genetic disorder which has several phenotypic features including craniofacial anomalies, cardiac, vertebral and central nervous system defects and was first described in 1952 by Goldenhar [1, 2].

The clinical presentation may be mild or severe and comprises signs of mutiple organ systems in a wide spectrum. Central nervous system abnormalities, auricular and ocular deformities, facial asymmetry, facial clefts, vertebral anomalies, renal and cardiac anomalies were reported [3, 4]. Cardiovascular anomalies include ventricular septal defects (VSD), atrial septal defects (ASD), patent ductus arteriosus (PDA), Tetralogy of Fallot (ToF), pulmonary atresia with VSD, double outlet right ventricle (DORV), aberrant right subclavian artery, coarctation of aorta, transposition of the great arteries (TGA), double inlet left ventricle, cor triatriatum, pulmonary artery stenosis, aortic stenosis, persistent left superior vena cava, partially or totally abnormal pulmonary venous return and bicuspid aortic valve [5–10].

Persistent fifth aortic arch (PFAA), is a rare cardivascular anomaly first described in 1969 by Van Praagh and usually associate other cardiac defects [11]. The pathology has four types according to Freedom et al.; a) Type 1, systemic-to- systemic, b) Type 2: systemic-to-pulmonary, c) Type 3: pulmonary-to-systemic and d) Type 4: bilateral [12]. In this case we present a Type 1 PFAA, also named as double lumen aortic arch, as a component of OAVS and this is the first case reported in association with this genetic disorder according to the literature review.

## Case presentation

A 7 month old male patient was referred to our medical centre with symptoms of respiratory distress due to choanal atresia. An operation plan was made for this disorder when he was 48 days old. He was born by normal spontaneous vaginal delivery at 34th gestational week with a birth weight of 1750 g. He had no siblings. The non-consanguineous parents were both 23 years old. The mother had no medical history for spontaneus or medical abortus. The family history for congenital heart defect was unremarkable.

**Fig. 1:**
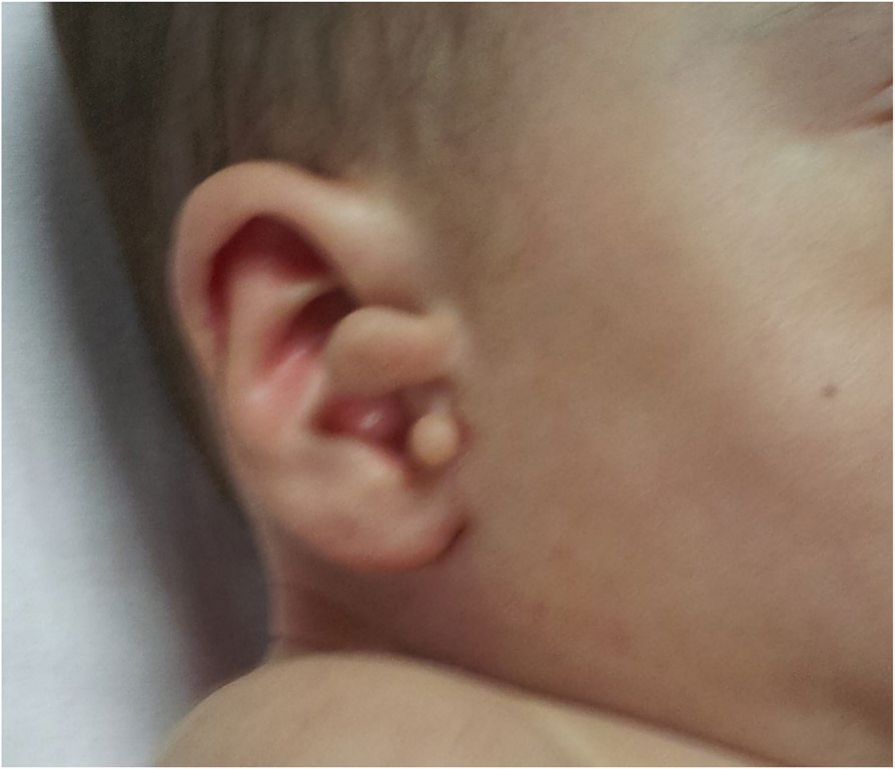
Preauricular skin tags

**Fig. 2:**
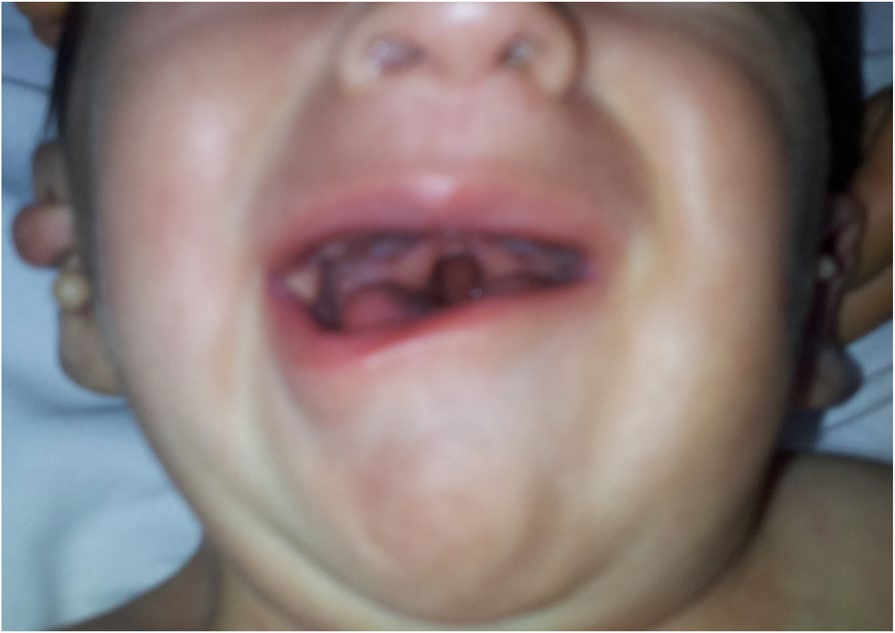
Facial asymmetry and cleft palate

**Fig. 3:**
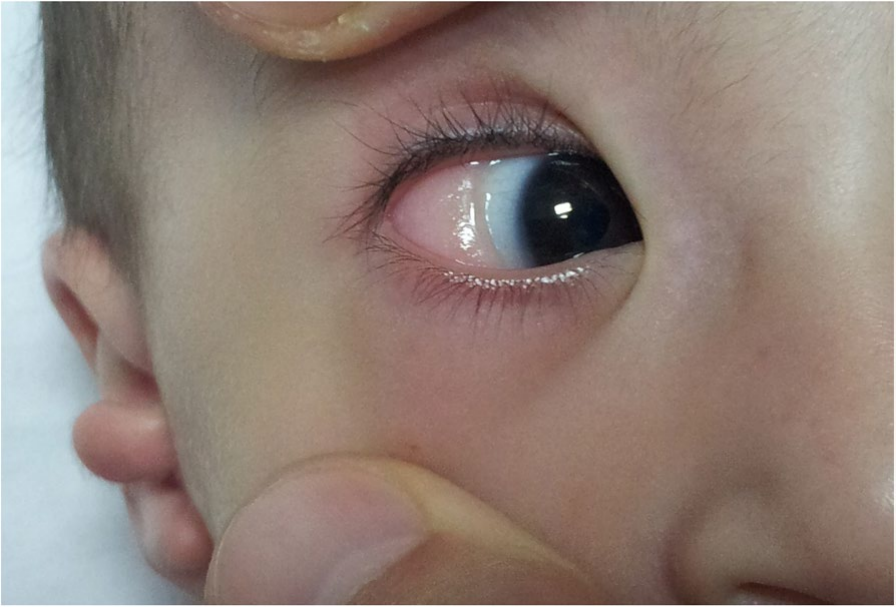
Epibulbar dermoid cycst

The patient weighed 5.5 kg (< 3 percentile) and his height was 61 cm (< 3 percentile). Vital signs were normal. Blood pressures were in normal range and there was no significant blood pressure gradient between upper and lower extremities, with systolic pressures 75 mmHg and 77 mmHg respectively. Physical examination revealed dysmorphic appearance, facial asymmetry (absence of depressor anguli oris muscle), short neck, choanal atresia, cleft palate, bilateral preauricular skin tags, bilateral hypoplastic ear lobes, epibulbar dermoid cyst in right eye (Figs. 1, 2 and 3). On cardiac examination there was a 3/6 degree holosystolic heart murmur best heard on left middle side of sternum. The other organ system examinations were all normal.

Routine blood tests including complete blood count, blood biochemistry, arterial blood gas, coagulation tests were all in normal ranges. Abdominal ultrasonography was normal.

Roentgenograms revealed hypoplasia of the right first rib, agenesis of left 12th rib, hypoplasia of the left side of 5th thoracic vertebra, unilateral 3rd thoracic hemi-vertebra and bilateral hemi-vertebra sign on 5th thoracic vertebra.

The paranasal computed tomography revealed bilateral choanal atresia, osseous in right side and membranous in left side on coronal and axial sections.

Cranial magnetic resonance imaging revealed increased extra cerebral cerebrospinal fluid distance of fronto-temporal and pre-pontin regions. Third and lateral ventricles were mildly dilated. Cleft palate was also noted in coronal sections.

**Fig. 4:**
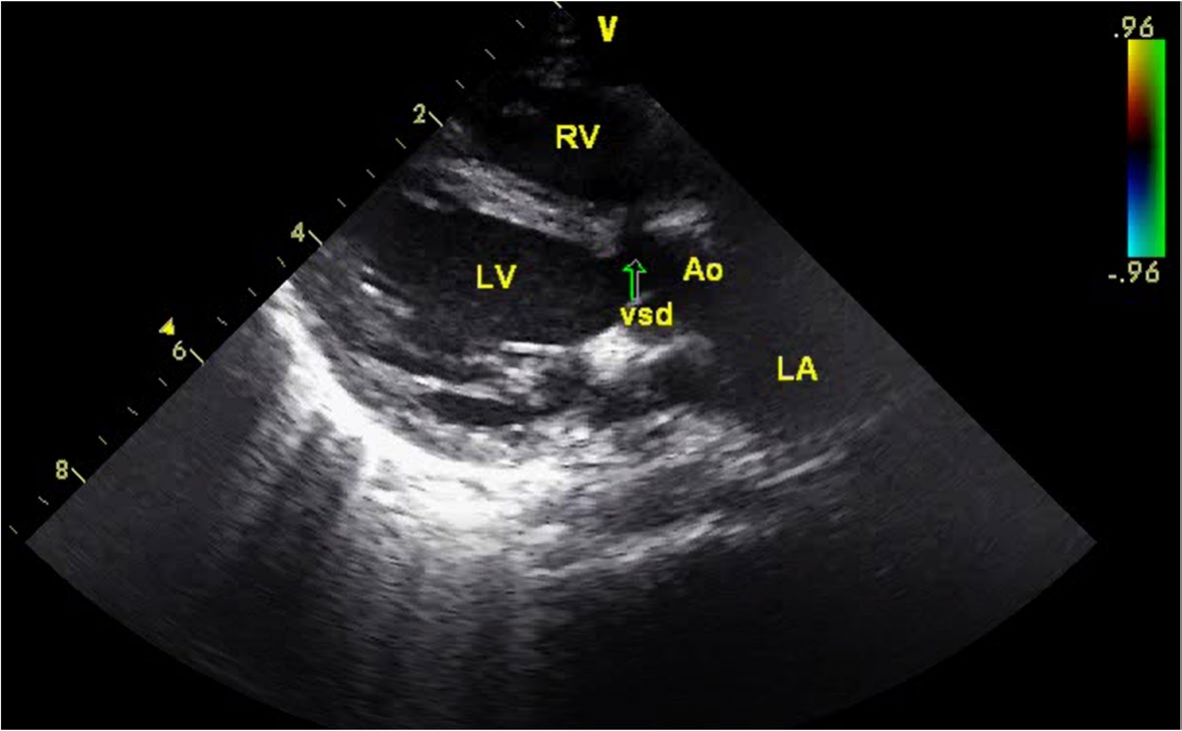
VSD echocardiographic view

**Fig. 5:**
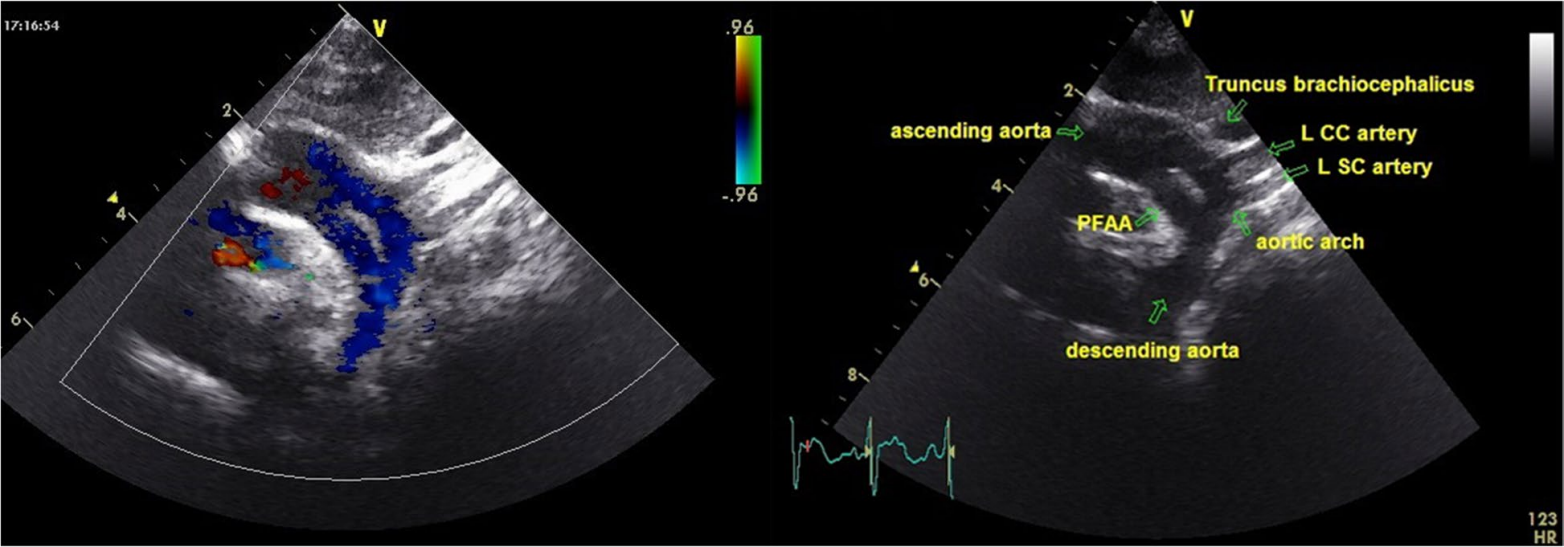
Persistent fifth aortic arch; echocardiographic 2D and color doppler view

In echocardiographic assessment a VSD on perimembraneous outlet septum - 5.5 mm in diameter and with left to right shunt- (Fig. 4), a small secundum ASD and a double lumen appearance in transverse aortic arch were determined. Brachiocephalic trunk, left common carotis artery and left subclavian artery were seperating from upper, original aortic arch (Fig. 5). Measurements revealed aortic valve annulus 9 mm, ascending aorta 10 mm, upper aortic arch 4.6 mm, lower arch –PFAA- 5.8 mm, proximal descending aorta 5 mm. Systolic functions were in normal range with ejection fraction 69% and shortening fraction 37%. Left ventricle was mildly dilated, with end-diastolic diameter of 26 mm (Z score + 1.8 SD). Pulmonary artery was dilated (diameter 15.6 mm, Z score + 3.1 SD). There was no subvalvular or valvular pulmonary stenosis.

The ECG findings were in normal ranges with sinus rhythm, a heart rate of 135 beats per minute, normal axis, normal intervals including corrected QT of 0.39 s. There was no pathological ST or T wave findings, AV block or extra beats.

**Fig. 6:**
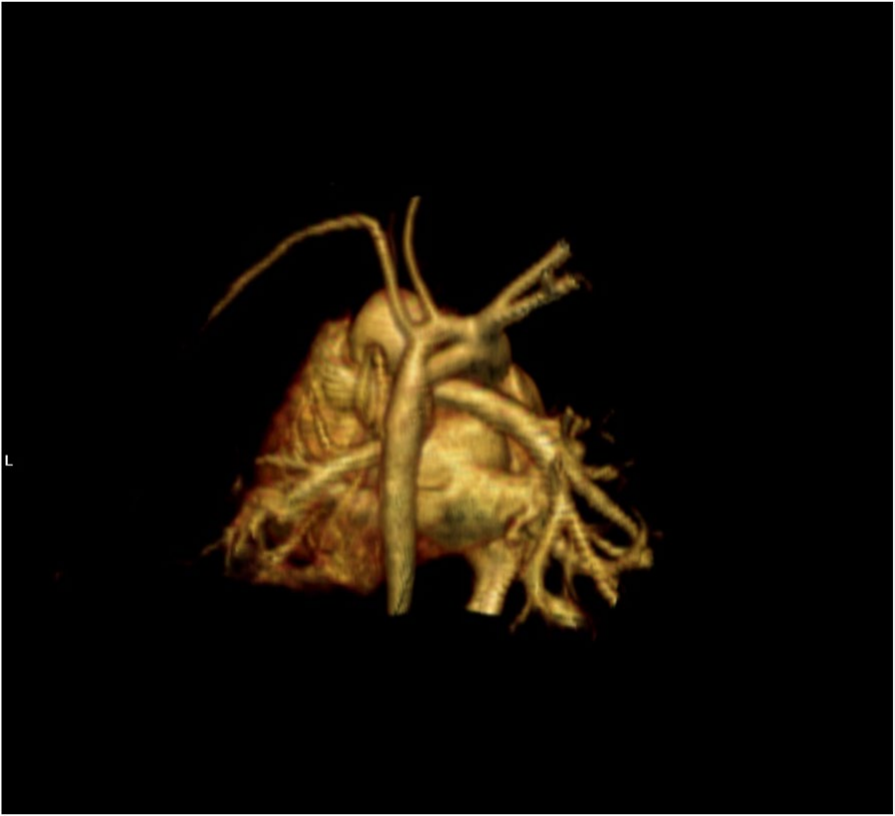
Persistent fifth aortic arch; CT view

In three-dimensional computed tomographic angiography it was determined that, aortic arch was divided into two parallel lumens opposite to the seperation of right brachiocephalic truncus, suggesting persistent fifth aortic arch, and these parallel lumens joined with the proximal part of the descending aorta. Left carotis communis artery and left subclavian artery were leaving from original, superior, aortic arch as its branches (Fig. 6).

Routine chromosomal analysis of peripheral blood showed normal male constitutional caryotype 46, XY and fluorescense in situ hybridization (FISH) analysis for 22q11.2 deletion (Vysis Inc.) was also normal.

**Fig. 7:**
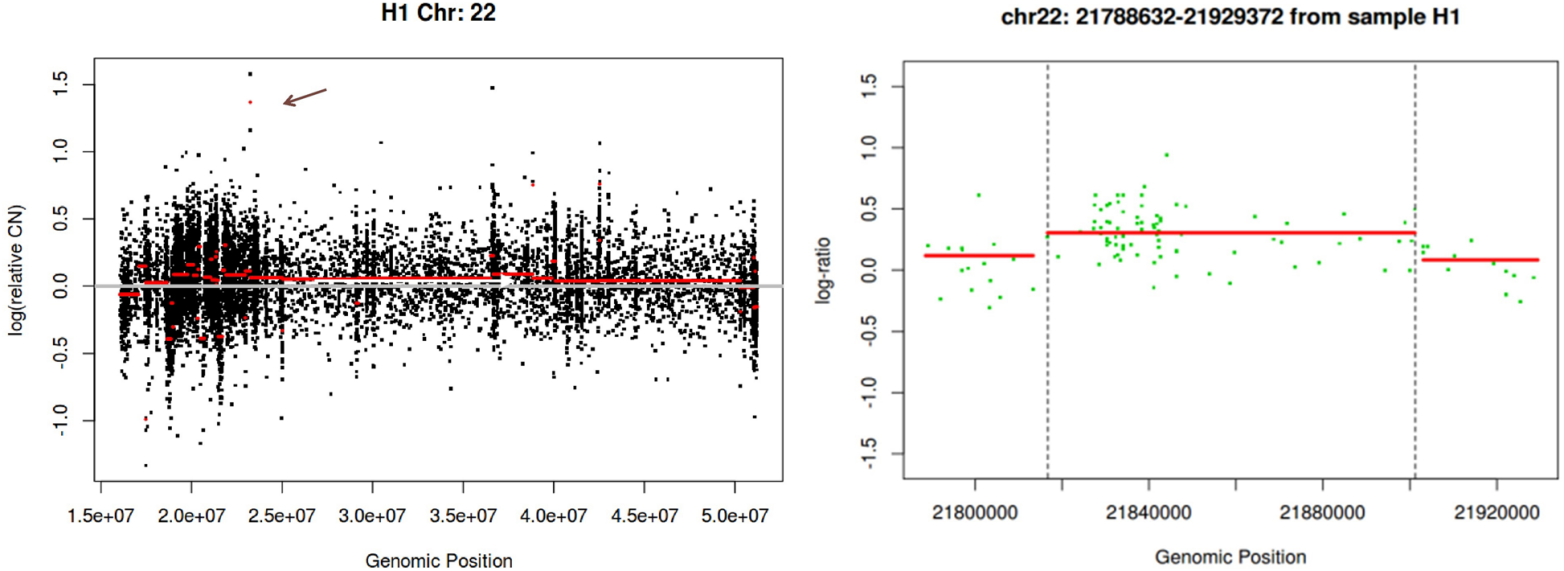
Array study

As the patient presented strong facial dysmorphism and severe congenital heart defects, an Array CGH analysis was planned to rule out the possible microdeletions and duplications. This was done by NimbleGen CGH 6x630K ISCA Plus Cytogenetic Arrays after obtaining a normal karyotype and normal FISH results for 22q11.2. Array comparative genomic hybridization (aCGH) analysis also did not reveal any apparent causative mutation. However a 84 kb duplication in the region 22q11.2 was interpreted as possibly pathogenic (Figs. 7). The mutation was related to Cat Eye Syndrome which included overlapping clinical manifestations with OAVS. Detailed genetic study could not be performed for the parents because of social reasons.

The patient was diagnosed as OAVS, operated for bilateral choanal atresia when he was 50 days old with a satisfactory result. Cardiac glycoside and diuretic medical treatment were given in clinical follow up at fifth months of age because of clinical symptoms of heart failure as insufficient weight gain and mild continuous tachypnea. Chronic heart failure symptoms were under control with this treatment, he gained adequate weight and tachypnea improved within 1 month. He had no extra symptoms due to double lumen aorta. He is still under clinical follow up and gaining adequate weight.

## Discussion and conclusions

Cat Eye syndrome (CES), CHARGE syndrome (coloboma, heart anomaly, atresia choanal, retardation, genital and ear anomalies) and OAVS are three major clinical entities with similar clinical findings inluding congenital heart anomalies; ear and eye anomalies, gastrointestinal system malformations, rib and vertebrae anomalies and growth and motor retardation. Due to the association of preauricular tags, epidermoid eye cyst, bifid uvula and rib and vertebrae anomalies, it can be apparently discussed that the present case is to be differentiated mainly between these three syndromes.

Individuals with CES, frequently have colobomas, downslanting eyelid folds, widely spaced eyes, misshapen ears and preauricular tags and pits, and/or absence of the anal canal. Additional features may commonly include variable congenital heart defects, renal abnormalities, and skeletal defects. The critical region for CES is currently estimated to be about 2 Mb and contains at least 14 genes [13]. Duplications of this region may cause ocular coloboma, anorectal, urogenital and congenital heart malformations and ear anomalies. Knijnenburg et al. described a family in which a 600 kb intrachromosomal triplication is present in at least three generations. The family members showed anal atresia and preauricular tags or pits, which match part of the phenotype of this syndrome [14]. Array comparative genomic hybridization data revealed that our patient does not cover the critical region of CES in spite of the duplicated region obtained on chromosome 22q11.2. The 84 kb duplication in our patient was out of the above mentioned critical region.

CHARGE syndrome is characterized by a group of congenital malformations including coloboma of the eye; heart defects; choanal atresia, growth and psychomotor retardation, genital and ear anomalies. Four or more of these characteristic features must be present for a diagnosis of CHARGE association. Other findings, such as cleft palate/lip, swallowing difficulties, facial palsy, tracheoesophageal fistula and renal malformations are commonly associated. In most cases, CHARGE association appears sporadically; however, some familial cases have been reported. CHARGE syndrome is caused by heterozygous mutation in the CHD7 (608892) on chromosome 8q12 and by mutations at 7q21. Array comparative genomic hybridization data for our case did not show any mutation at the relevant CHARGE syndrome regions.

Oculoauriculovertebral spectrum is a genetic disorder with highly variable phenotypes including craniofacial, vertebral, auditory, ocular, central nervous system and cardiac anomalies. Although most cases are sporadic, some familial cases were reported with autosomal dominant or autosomal recessive inheritance [15, 16]. Due to clinical manifestations reported in detail above, our patient was considered to present an OAVS case. Among OAVS cases several microdeletions including 5q13.2, 5pter-p15.33 and 22q11.2 were reported [17–20]. Our case had normal male karyotype, 46, XY. As he had conotruncal heart defects fluorescence in situ hybridisation (FISH) analysis was performed and no 22q11 deletion was found. However, a microduplication on 22q11.2 was detected. Microduplications on 22q11.2 has a wide pheotypic spectrum including mental retardation, facial anomalies and congenital heart defects [21]. Ensenauer et al. considered the microduplications on 22q11.2 as a new syndrome and evaluated findings of 13 patients. Two of these patients had congenital heart defects, one ToF and the other hypoplastic left heart syndrome (HLHS) with aortic arch interruption [22]. McMahon et al. reported a patient with 22q11.2 microduplication and HLHS and hypoplastic pulmonary arteries [23]. Sparkes et al. also reported two patients with ToF and HLHS and microduplications on chromosome 22q11.2 [24] With these reports we consider that 22q11.2 microduplications may be a probable cause of congenital heart defects; but the association with OAVS is not verified yet.

Hemifacial microsomia causes facial asymmetry and is presented by most of the patients [15]. Our patient had also facial asymmetry due to unilateral absence of depressor anguli oris muscle. Oro-cranio-facial malformation, microsomia, macrostomia, cleft lip and/or palate, bifid uvula and epiglottic fold malformation can be seen in these patients [6]. The patient had bifid uvula.

Auricular deformities comprise microtia, deafness, preauricular skin appendages and fistulae [3, 6, 15]. The patient presented external auditory canal deformity and preauricular skin tags.

The most common ocular deformities are epibulbar dermoids, lipodermoids or coloboma. Microcornea, microphthalmia, anaophthalmia, iris atrophy and cataract can also be seen [3, 6, 15]. There was epibulbar dermoid cyst in right eye of our patient.

Vertebral anomalies include hemivertebrae, atlas anomalies, spina bifida [22]. On X-ray scan of our case hypoplasia of the right 1th rib, agenesis of left 12th rib, hypoplasia of the left side of 5th thoracic vertebra, unilateral hemi-vertebra sign on 3th thoracic vertebra and bilateral hemi-vertebra sign on 5th thoracic vertebra were seen.

Central nervous system (CNS) anomalies are components of OAVS. Cerebral hypoplasia, corpus callosum dysgenesis, dilated lateral cerebral ventricles (asymptomatic hydrocephalus), frontal hypodensities, intracerebral calsifications, intracranial teratomas and cranium defects were reported before [4, 25]. As CNS anomalies, our patient had increased extra cerebral cerebrospinal fluid distance of fronto-temporal and pre-pontin regions and mildly dilated third and lateral ventricles suggesting hydrocephaly.

A wide variety of cardiac anomalies were reported to be associated with OAVS but no single defect is characteristic. Septal defects and ToF are frequent. Transposition of the great arteries, hypoplasia of the aortic arch with mild coarctation of the aorta, PDA, pulmonary stenosis, dextrocardia, double outlet right ventricle, and other aortic arch abnormalities have been described [26]. Strömland et al. reported VSD as most frequent cardiac defect [6]. Rosa et al. found cardiac defects in 39.4% of their patients and they reported ToF, intraventricular communication with pulmonary atresia, TGA, secundum ASD, atrioventricular septal defect, PDA, pulmonary artery stenosis and cor triatriatum as cardiac defects [8]. Our patient had VSD and secundum ASD. Nakajima et al. reported a 54 years old patient with partial anomalous pulmonary venous connection, PDA, an anomalous origin of the coronary arteries, a right-sided descending aorta, and Wolf Parkinson White syndrome [27]. The electrocardiography of our patient revealed no abnormalities or arrhythmias. Digilio et al. evaluated a wide series of OAVS with 87 patients and found that 32% of the patients had cardiac defects. Most frequent defects were outflow/conotruncal defects (39%) including ToF, DORV, pulmonary atresia with VSD, TGA, coarctation of aorta and double aortic arch [10]. In the case we are presenting, there was an aortic arch anomaly described as double lumen aorta or persistent fifth aortic arch.

Persistent fifth aortic arch is a rare malformation of the aorta and usually associated with other cardiovascular anomalies. Clinically it has two main forms which reveal systemic-to-systemic or systemic-to-pulmonary connections. The anomalous arch can connect the ascending aorta to the descending aorta or as in the systemic-to-pulmonary type, a connection between the ascending aorta and a derivative of the 6th arch, usually the left pulmonary artery may occur. The clinical signs and severity of this anomaly change via associated cardiovascular anomalies and which of these anatomic connections exists. Ventricular septal defects and ToF are the most common associated anomalies. It may be in association with PDA, coarctation of aorta or truncus arteriosus [28–32]. Akhfash et al. presented a case with associated coronary artery anomaly [33]. Lee et al. reported cases with persistent fith aortic arch in association with 22q11 deletion [34, 35]. As relationship between 22q11 deletion and aortic arch anomalies is well known, we performed FISH analyse to our patient and found no deletion.

Diagnosis of Type 1 PFAA can be easily done with non-invasive imaging methods as echocardiography, computed tomography and magnetic resonance imaging [33, 36, 37]. It can be shown by conventional angiography but it is not necessary if there is no other indication for angiography. In echocardiography as in our case, two parallel lumens of the aorta can be seen in suprasternal view, one is the ascending aorta and the other is the lumen of the PFAA. We did not perform a conventional angiography to our patient because his symptoms of chronic heart failure due to VSD is under control with diuretic and cardiac glycoside treatment. The patient does not currently require surgical treatment, but surgery will probably be planned in follow up, for VSD closure and maybe a simultaneous surgical intervention for the aortic arch.

In conclusion; Oculoauriculovertebral (OAVS) spectrum also known as Goldenhar Syndome is a heterogeneous disorder and has a wide phenotypical spectrum with associated anomalies of various organs. Overlapping clinical features with Cat Eye Syndrome (CES) and CHARGE syndrome cause clinical diagnosis to be relatively difficult. The aCGH analysis can be a method of choice for determining cryptic chromosomal inbalances. Cardiovascular anomalies frequently accompany OAVS. We present a new association, PFAA with OAVS and to the best of our knowledge this is not reported before.

## Data Availability

The datasets used and/or analysed during the current study are available from the corresponding author on reasonable request.

## Abbreviations

OAVS: Oculo-auriculo-vertebral spectrum
MIM: Mendelian Inheritance in Man
VSD: Ventricular septal defect
ASD: Atrial septal defects
PDA: Patent ductus arteriosus
ToF: Tetralogy of Fallot
PFAA: Persistent fifth aortic arch
CNS: Central nervous system
SD: Standard deviation
ECG: Electrocardiography
HLHS: Hypoplastic left heart syndrome
